# A species distribution model of the giant kelp *Macrocystis pyrifera*: Worldwide changes and a focus on the Southeast Pacific

**DOI:** 10.1002/ece3.10901

**Published:** 2024-03-01

**Authors:** Daniel Gonzalez‐Aragon, Marcelo M. Rivadeneira, Carlos Lara, Felipe I. Torres, Julio A. Vásquez, Bernardo R. Broitman

**Affiliations:** ^1^ Doctorado en Ciencias, mención en Biodiversidad y Biorecursos, Facultad de Ciencias Universidad Católica de la Santísima Concepción Concepcion Chile; ^2^ Instituto Milenio en Socio‐Ecología Costera (SECOS) Santiago Chile; ^3^ Núcleo Milenio UPWELL; ^4^ Centro de Estudios Avanzados en Zonas Áridas Coquimbo Chile; ^5^ Departamento de Biología Marina, Facultad de Ciencias del Mar Universidad Católica del Norte Coquimbo Chile; ^6^ Departamento de Ecología, Facultad de Ciencias Universidad Católica de la Santísima Concepción Concepcion Chile; ^7^ Centro de Investigación en Recursos Naturales y Sustentabilidad Universidad Bernardo O'Higgins Santiago Chile; ^8^ Data Observatory Foundation, ANID Technology Center No. DO210001 Santiago Chile; ^9^ Centro de Investigación y Desarrollo Tecnológico en Algas y Otros Recursos Biológicos (CIDTA) Coquimbo Chile; ^10^ Facultad de Artes Liberales Universidad Adolfo Ibañez Viña Del Mar Chile

**Keywords:** climate change, distribution model, habitat forming, kelp forests, projection, Southeast Pacific

## Abstract

Worldwide climate‐driven shifts in the distribution of species is of special concern when it involves habitat‐forming species. In the coastal environment, large Laminarian algae—kelps—form key coastal ecosystems that support complex and diverse food webs. Among kelps, *Macrocystis pyrifera* is the most widely distributed habitat‐forming species and provides essential ecosystem services. This study aimed to establish the main drivers of future distributional changes on a global scale and use them to predict future habitat suitability. Using species distribution models (SDM), we examined the changes in global distribution of *M*. *pyrifera* under different emission scenarios with a focus on the Southeast Pacific shores. To constrain the drivers of our simulations to the most important factors controlling kelp forest distribution across spatial scales, we explored a suite of environmental variables and validated the predictions derived from the SDMs. Minimum sea surface temperature was the single most important variable explaining the global distribution of suitable habitat for *M*. *pyrifera*. Under different climate change scenarios, we always observed a decrease of suitable habitat at low latitudes, while an increase was detected in other regions, mostly at high latitudes. Along the Southeast Pacific, we observed an upper range contraction of −17.08° S of latitude for 2090–2100 under the RCP8.5 scenario, implying a loss of habitat suitability throughout the coast of Peru and poleward to −27.83° S in Chile. Along the area of Northern Chile where a complete habitat loss is predicted by our model, natural stands are under heavy exploitation. The loss of habitat suitability will take place worldwide: Significant impacts on marine biodiversity and ecosystem functioning are likely. Furthermore, changes in habitat suitability are a harbinger of massive impacts in the socio‐ecological systems of the Southeast Pacific.

## Graphical abstract

We modeled the global distribution of giant kelp, *Macrocystis pyrifera*, with projection under climate change scenarios. The results are sobering and provide a starting point for future modeling exercises that will be able to draw on a more comprehensive set of physical and ecological drivers that are currently unavailable to the broader community.

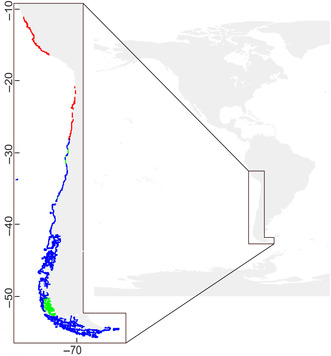

## 1 INTRODUCTION

Biogeographic‐scale species range shifts are globally increasing due to climate change driven by human activities (Burrows et al., 2011; Masson‐Delmotte et al., 2021). Marine ecosystems are changing rapidly following widespread changes in the abundance and distribution of a wide range of species (Edgar et al., 2023; Hoegh‐Guldberg & Bruno, 2010). Forest‐forming laminarian algae, kelps, contribute with key ecosystem services and due to their role as ecosystem engineers sensu Jones et al. (1994), and changes in their distribution are of special concern under climate change (Babcock et al., 2019; Cuba et al., 2022; Fragkopoulou et al., 2022; Steneck et al., 2002; Thomsen et al., 2010). Underwater kelp forests provide complex three‐dimensional habitats, support exceptionally high rates of primary productivity, and maintain diverse and productive food webs that represent important conservation and management goals (Hastings et al., 2007; Reed & Brzezinski, 2009; Steneck et al., 2002). They are found throughout the world, dominating approximately 25% of the coastlines (Steneck et al., 2013), and are widespread in cold, temperate, and polar waters, as the main factor controlling the distribution of the kelp is the temperature of the seawater (Fragkopoulou et al., 2022; Krumhansl et al., 2016; Lüning, 1991). Due to the importance of temperature, ongoing climate change is altering and is expected to strongly modify the distribution of kelp in the future (Davis et al., 2022; Smale, 2020; Steneck et al., 2002). The loss of kelp forests has already been reported in multiple areas of the world as a result of rising local temperatures (Butler et al., 2020; Cavanaugh et al., 2019; Filbee‐Dexter et al., 2016; Krumhansl et al., 2016), and further changes have been forecast for the group as a whole using species distribution models (Assis et al., 2016, 2017; Fragkopoulou et al., 2022; Sudo et al., 2020). Changes in kelp abundance drive the reconfiguration of the community primarily through changes in kelp stipe biomass and retention that translates into a loss of associated taxa (Teagle & Smale, 2018). Therefore, the study of the effect of climate change and the increase in ocean temperature on habitat‐forming kelp species remains a research priority.

Among all genera of kelp, *Macrocystis pyrifera* (Linnaeus) C. Agardh, 1820 or giant kelp, is the most widely distributed kelp species with an amphiequatorial pattern spanning the temperate eastern Pacific coasts, the Southwestern Pacific (New Zealand), the Southeast Indian Ocean (Australia), the Southern coasts of the Atlantic (Argentina and South Africa), and most of its circumantarctic islands (Graham, Vasquez, & Buschmann, 2007). Giant kelp appears to have evolved in the Northern Hemisphere and crossed the equator through deep water refuges (Graham, Kinlan, et al., 2007; Silberfeld et al., 2010). Recently, further evidence for this theory was added through a phylogeographic study showing that the Northern Hemisphere has a significantly higher genetic diversity (Assis et al., 2023). Ecological plasticity is a key factor for the global success of *M*. *pyrifera*: both gametophytes and sporophytes can settle in different rocky substrata and under varied environmental conditions, become established, and complete their life cycle in less than a year (Buschmann et al., 2006). Dispersal can also take place through rafting, which allows connectivity over large spatial scales (Bernardes Batista et al., 2018; Rothäusler et al., 2011). If conditions are favorable, they can persist for several seasons and form multigenerational stands (Dean et al., 1989).

At different stages of its life cycle, temperature drives dispersal, settlement, development, and consequently the global distribution of giant kelp is restricted to a thermal range between 4 and 20°C (Schiel & Foster, 2015). Genetic differences between populations in conjunction with local adaptations to thermal stress suggest that different populations may have different thresholds according to their local conditions (Hollarsmith et al., 2020; Kopczak et al., 1991). On the other hand, other studies did not find differences in the physiological response to ocean warming and canopy loss due to heatwaves between different populations, thus advancing the hypothesis of an absolute tolerance threshold to temperature, beyond which local adaptation is no longer effective and leads to local loss of *Macrocystis* forests (Cavanaugh et al., 2019; Fernández et al., 2021). Temperature can also exhibit a strong inverse correlation with nitrate concentration in the water column, particularly in upwelling ecosystems (Nielsen & Navarrete, 2004; Palacios et al., 2013). These two factors strongly affect the populations of *Macrocystis*: temperatures >23°C and nitrate concentrations <1 μmol L^−1^ can lead to severe reductions in canopy biomass and blade elongation rates (Rodriguez et al., 2016; Zimmerman & Kremer, 1984). Less studied is the case of salinity, but effects on zoospore release, germination, and early growth have been observed, with greater success at higher salinity, as well as a wider range of tolerance for salinity in populations in estuarine environments, for example, Southern Chile (Buschmann et al., 2004).

Variations in the abundance of *Macrocystis* kelp forests have been observed in different parts of the world with very diverse trends (Smale, 2020). Recently, the use of remote sensing data showed that long‐term and low‐frequency marine heat waves associated with climate change may be driving trends in kelp biomass along the Northeast Pacific (Bell et al., 2020; Cavanaugh et al., 2019). The distribution of giant kelp in Australia is changing; future increases in temperature are likely to result in changes in the edge of the equator range and a reconfiguration of the associated community (Wernberg et al., 2011). In Tasmania, a decline in the extent of *M*. *pyrifera* associated with changes in physical conditions such as increasing sea temperature has caused a cascade of ecological changes (Butler et al., 2020; Johnson et al., 2011). In addition, a loss of canopy area has been observed since 2017 in the Falkland Islands, reaching the minimum area observed in the last three decades by remote sensing (Houskeeper et al., 2022). More studies of this habitat‐forming macroalgae species are key to further elucidate the different trends over local, regional, and global scales.

*Macrocystis pyrifera* is one of the most representative macroalgae species in low intertidal and subtidal areas of the Southeast Pacific, but little is known about the local distribution of the patches of giant kelp forest (Aguilera et al., 2019; Avila‐Peltroche & Padilla‐Vallejos, 2020). In Peru, the northern distribution limit has recently been reported to be in Lima (12° S) (Carbajal Enzian & Gamarra, 2018). Although it seems to be the consequence of a range contraction—it has been reported further equatorward (4° S) in the middle of the twentieth century (Juhl‐Noodt, 1958), which coincides with other authors who placed the beginning of the distribution range of giant kelp in the Southeast Pacific at 6° S (Buschmann et al., 2004). The *M*. *pyrifera* populations are distributed along the coast of Chile, where in the north and center the intense harvesting of natural populations has affected the entire ecosystem (Buschmann et al., 2014; Vásquez, 2016). The extraction of *M*. *pyrifera* in Chile is increasing, 31,860.3 tons were extracted on average per year between 2012 and 2021 in Chile (a total of 318,603 tons) (SERNAPESCA, 2021). On the contrary, remote populations in Patagonia and Tierra del Fuego, where human impact is very slight, have remained persistent for at least the last 200 years (Friedlander et al., 2020; Mora‐Soto et al., 2021). Both countries, Peru and Chile, are investing, promoting, and developing giant kelp aquaculture as an alternative to the exploitation of natural stocks, which may also be affected due to ongoing climate change (Avila‐Peltroche & Padilla‐Vallejos, 2020; Buschmann et al., 2014).

By linking the occurrence of important habitat‐forming species with climatic variables and the effects of global change on their predicted distribution, Species Distribution Models (SDMs) are powerful tools for conservation and management (Austin, 2002; Robinson et al., 2017). Despite the widespread use of SDMs in terrestrial species, marine ecosystems have received limited attention (Melo‐Merino et al., 2020; Robinson et al., 2011). Following the advent of widely available and accessible remote‐sensing information, the tide has started to turn, particularly for coastal ecosystems (Melet et al., 2020). Following the importance of kelp forests in coastal ecosystems, several SDM studies in the last decade have predicted changes in the distribution of these species (Assis et al., 2017; Castro et al., 2020; Martínez et al., 2018; Sudo et al., 2020), highlighting the loss of habitat suitability in the low‐latitude section of the species range, which is sometimes compensated for by expansions to higher latitudes (Assis et al., 2016; Assis, Araújo, & Serrão, 2018; Davis et al., 2022). A global distribution model of the kelp biome estimated that all kelps occupy 2,033,936 km^2^ (36% of the world's coastline), making it the most widely distributed in marine habitat suitability (Jayathilake & Costello, 2020, 2021). The global SDM study included the 18 species that form kelp forest ecosystems; therefore, distribution models for *M*. *pyrifera* remain limited to regional scales. For example, Martínez et al. (2018) suggested the extinction of giant kelp in Australia by 2100 and widespread regional range shifts (Graham, Vasquez, & Buschmann, 2007; Smale, 2020). The aim of our study was to examine the global distribution of suitable habitat for *M*. *pyrifera* under different global change scenarios by selecting and using a narrow set of key environmental variables to focus on the poorly studied Southeast Pacific coast. We hypothesized that predicted changes in oceanographic conditions will drive regional shifts in the suitable habitat for *M*. *pyrifera* leading to the extirpation of the species over a large part of its current range with potentially large socio‐ecological impacts.

## 2 METHODS

### 2.1 Species occurrence data

**FIGURE 1:**
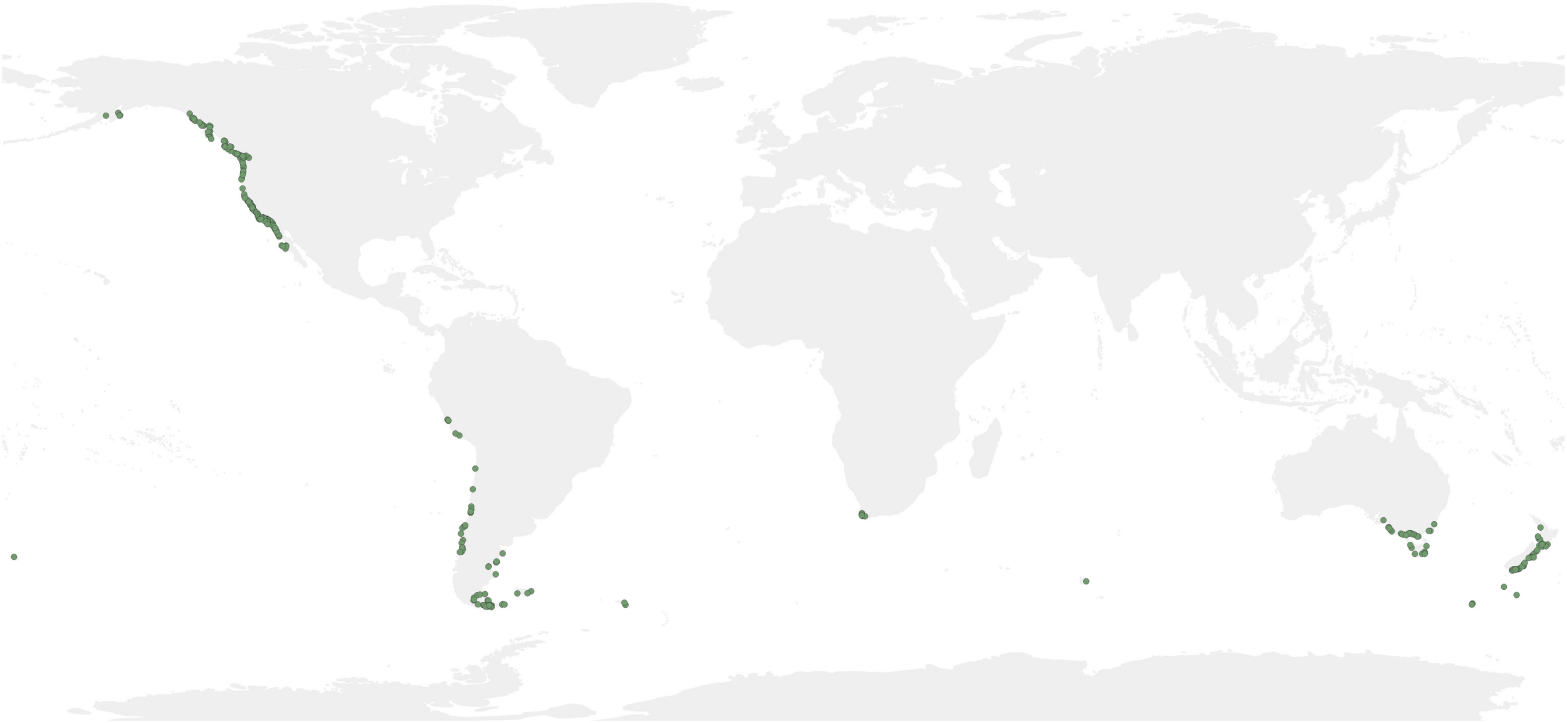
Global occurrences of *Macrocystis pyrifera* since 1970 downloaded from GBIF and used in this study. The occurrences were clean and a subsample were selected by creating a grid of 9.2 km^2^ and randomly sampled one occurrence per grid cell to reduce the spatial aggregation of records.

To map the occurrence of giant kelp worldwide, we took advantage of the capabilities of the Global Biodiversity Information Facility (GBIF) and extracted a total of 40,349 occurrences from its open access database on February 28, 2022 (GBIF.org, 2022). The occurrences were also downloaded from Ocean Biodiversity Information System (OBIS), but after crossing both databases, all occurrences were already in GBIF, with the latter being more complete. First, we filtered all occurrences prior to 1970, unreliable records falling on land, coordinates that were duplicated and with a positional uncertainty >10 km (Feng, Park, Walker, et al., 2019). Then a subsample of the records was selected by creating a 9.2 km^2^ cell size grid to randomly sample one *M*. *pyrifera* occurrence per grid cell to reduce spatial aggregation, ensuring a homogeneous density of records throughout the study area (Fourcade et al., 2014). The size of the grid was chosen to ensure that the resolution of the environmental variables was similar to or lower than the spatial resolution of the species records (Barbosa et al., 2010). Finally, after filtering the data, we kept 366 occurrences, which match the known historical distribution of Graham, Vasquez, and Buschmann (2007) (Figure 1).

### 2.2 Environmental predictors

We obtained marine environmental predictors with a resolution of 5 arcminute, approximately 9.2 km at the equator, from Bio‐Oracle v2.2 and Global Marine Environment Datasets (GMED) (Assis, Tyberghein, et al., 2018; Basher et al., 2018; Tyberghein et al., 2012). To select the physical and geomorphological conditions favored by *M*. *pyrifera*, we developed a coast mask in the model using a coastline layer of Natural Earth (http://naturalearthdata.com/), chiefly depth of light penetration and wave sheltering (Graham, Vasquez, & Buschmann, 2007). We chose a 10 km^2^ width for the coastal masking layer to conserve at least one pixel of predictor variables worldwide. The complex geomorphology in certain coastlines (e.g., South of Chile, Alaska, or Scotland) resulted in a disjointed area that did not match the occurrence data, so the layer was manually edited using QGIS v3.22 (QGIS Development Team, 2022) and satellite imagery as reference (Google Earth hybrid). The global coastline was reviewed and improved by creating polygons around areas of the mainland coastline and islands that were not initially represented correctly. *M*. *pyrifera* occurrences were checked to ensure that they fell within the extension of the coast mask.

The environmental predictors considered to construct the SDMs were Sea Surface Temperature (SST) minimum (°C, SST_min_), SST mean (C, SST_mean_), SST maximum (°C, SST_max_), benthic temperature (°C), phosphate (mol m^−3^), calcite (mol m^−3^), photosynthetically active radiation (E m^−2^ day^−1^), nitrate (mol m^−3^), dissolved molecular oxygen (mol m^−3^), silicate (mol m^−3^), salinity (PSS), current velocity (m^−1^), pH, diffuse attenuation (m^−1^), and iron (μmol m^−3^). All 15 variables were cropped using the coastal mask layer and then visually checked to confirm that they matched the coastline and *M*. *pyrifera* occurrences. These predictor layers were created from monthly averages for the period 2000–2014 (Assis, Tyberghein, et al., 2018). The available layer for the wave height from GMED did not meet our criteria of spatial resolution for the coastline and a large number of gridded observations fell outside our mask, so we decided to abandon this variable (Graham et al., 1997; Hepburn et al., 2007).

### 2.3 Model performance, evaluation, threshold, and projections

The present distribution of *M*. *pyrifera* was modeled using Maxent 3.4.1 software (Phillips et al., 2021), with the occurrences and variables mentioned earlier. Maxent is an SDM machine learning method using presence‐only data to estimate the probability distribution of maximum entropy (Phillips et al., 2006; Phillips & Dudík, 2008). We utilized the model with 100 replicate runs with cross‐validation and 1000 maximum iterations. A maximum of 10,000 background points were randomly selected from all grids without occurrences to consider them as a spectrum of the general available conditions (Phillips et al., 2004, 2006). The background points were chosen from the coast area described above to reflect the environmental conditions (Merow et al., 2013).

A preliminary Maximum Entropy Model (Maxent) was run with the 15 predictor variables discussed above to observe the contribution of each one to the model. In parallel to Maxent analysis, we measured collinearity between environmental variables using the variance inflation factor (VIF) and Spearman's correlation coefficient (*r*_*s*_) considering high collinearity when values exceeded 5 and 0.75, respectively (Dormann et al., 2013). Finally, we selected environmental variables using the contribution of each variable extracted from the preliminary Maxent model, *r*_*s*_ and VIF values, and references from the literature supporting the importance of specific environmental variables in the distribution of *M*. *pyrifera*. The present global distribution model was simulated using eight variables (see Table 1). The three SST variables were correlated in the collinearity analyses. However, since our study is global in scope and each SST variable has been shown to play a role in the life history of giant kelp and, as a result, a major contributing variable in other studies of *M*. *pyrifera* modeling (Jayathilake & Costello, 2020; Schiel & Foster, 2015), we decided to keep them in the model. In some cases, the inclusion of correlated variables may be justified if they are determinants of the distribution species (Sillero & Barbosa, 2021). The minimum and mean SST are closely related to the presence of nutrient‐rich upwelling waters that are essential for the development and growth of *Macrocystis* (Graham, Vasquez, & Buschmann, 2007; Narayan et al., 2010). On the other hand, maximum SST regulates its low‐latitude distribution limit through physical and ecological constraints (Edwards, 2004; Ladah et al., 1999).

**TABLE 1:** Contribution of the variables to the Maxent model of the present model (all predictors) and the present model (subset predictors) of *Macrocystis pyrifera*.

Variables	Present model %	Projection model %
${\mathrm{SST}}_{\min}$	39.4	51.2
${\mathrm{SST}}_{\text{mean}}$	22.2	22.8
Phosphate	18.8	–
${\mathrm{SST}}_{\max}$	11.4	20.4
Calcite	3.9	–
Nitrate	2.1	–
Silicate	1.3	–
Salinity	1	5.7

Abbreviations: max, maximum; min, minimum; SST, Sea Surface Temperature.

To model the projected distribution of kelp under climate change for the range of emission scenarios presented in the IPCC report (IPCC, 2014), we selected the four variables from the previous modeling exercise that were available in future projections: SST_min_, SST_mean_, SSt_max_, and salinity, which together accounted for 74% of the contribution to the previous model performance. The four environmental variables were obtained from Bio‐Oracle for Representative Concentration Pathways (RCPs) 2.6, 4.5, 6.0, and 8.5 for 2090–2100 (Assis, Tyberghein, et al., 2018). The Bio‐Oracle layers are based on atmosphere–ocean‐coupled general circulation models (CGCMs) provided by the CMIP 5, specifically CCSM4, HadGEM2‐ES, and MIROC5 (Assis et al., 2017). The area and range of suitable habitat for *M*. *pyrifera* on the different models were calculated using QGIS v3.22 (QGIS Development Team, 2022). Again, we estimate the collinearity for future predictors (Feng, Park, Liang, et al., 2019). VIF and $\;r_{S}$ values indicated that the three SST for the present and RCPs scenarios were also collinear. The three SST variables yielded similar correlation values between the three scenarios, so we decided to retain them for the model. We performed a jack‐knife test to assess how the four environmental predictors contributed to model training (Figure S1). To further ensure correct extrapolation of the model, the novelty values of the RCP scenarios 2.6, 4.5, 6.0, and 8.5 were calculated with the Multivariate Environmental Similarity Surface (MESS) analysis in Maxent. MESS indicates environmental dissimilarity with negative values and similarity with positive values (Elith et al., 2010). We did not find negative values for the model distribution (Figure S2).

We used 70% of the occurrence as training data and reserved the remaining 30% for testing the model (Phillips et al., 2006). The area under the curve (AUC) of the Receiver Operating Characteristic (ROC) was used to evaluate the accuracy of the model (Peterson et al., 2008). The ROC curve and AUC measure the fit of the true positive rate (sensitivity) and the true negative rate (specificity) or the ability to discriminate presences from absences in the distribution model (Lawson et al., 2014; Phillips et al., 2004). On presence‐only data, AUC compares occurrences with background points. Hence, correctly defining the study area of comparison is of particular importance to avoid increasing the probability that the background points correspond to the true absences (Merow et al., 2013). In this study, the comparison area was limited to our coastline mask to avoid inflating the AUC following a high ratio between the distribution of the species and the spatial extent of the study area (Lobo et al., 2008).

The global distribution of *M*. *pyrifera* contributed to a higher specificity, that is, the proportion of background points. Therefore, we selected the threshold of “Maximum sensibility plus specificity” to illustrate the suitable distribution (Liu et al., 2013). Present‐only data models that maximize the sum of sensitivity and specificity are equivalent to maximizing the vertical and diagonal distance at the ROC curve and maximizing the true skill statistic as a measure of accuracy (Liu et al., 2013). The continuous distribution prediction from Maxent was converted to binaries of the presence and absence of suitable habitat according to the threshold defined above. The Maxent‐given threshold values for the “Maximum sensibility plus specificity” threshold were 0.432 for the model with eight variables and 0.416 for projection modeling with four variables. Only pixels with values above the former thresholds were considered suitable for *M*. *pyrifera* in this study.

## 3 RESULTS

The SDM of *M*. *pyrifera* modeled for the present using the eight predictors yielded a high AUC value (0.959), the probability of discriminating between the predicted presence records from the background points. The projected model for current conditions accurately predicted global occurrences as measured with the suitability index threshold “Maximum test sensitivity plus specificity.” The model built using four predictors also produced a high AUC (0.950).

The ${\mathrm{SST}}_{\min}$ showed the highest contribution to both the present and future projection models, followed by the ${\mathrm{SST}}_{\text{mean}}$ (Table 1). Both models were run with the same parameters, only changing the environmental variables considered. Therefore, global habitat suitability differences between models arose from environmental variables that were not considered in both: phosphates, calcite, nitrate, and silicate. Among the latter, phosphates made the greatest contribution to the model with 18.8% (Table 1).

**FIGURE 2:**
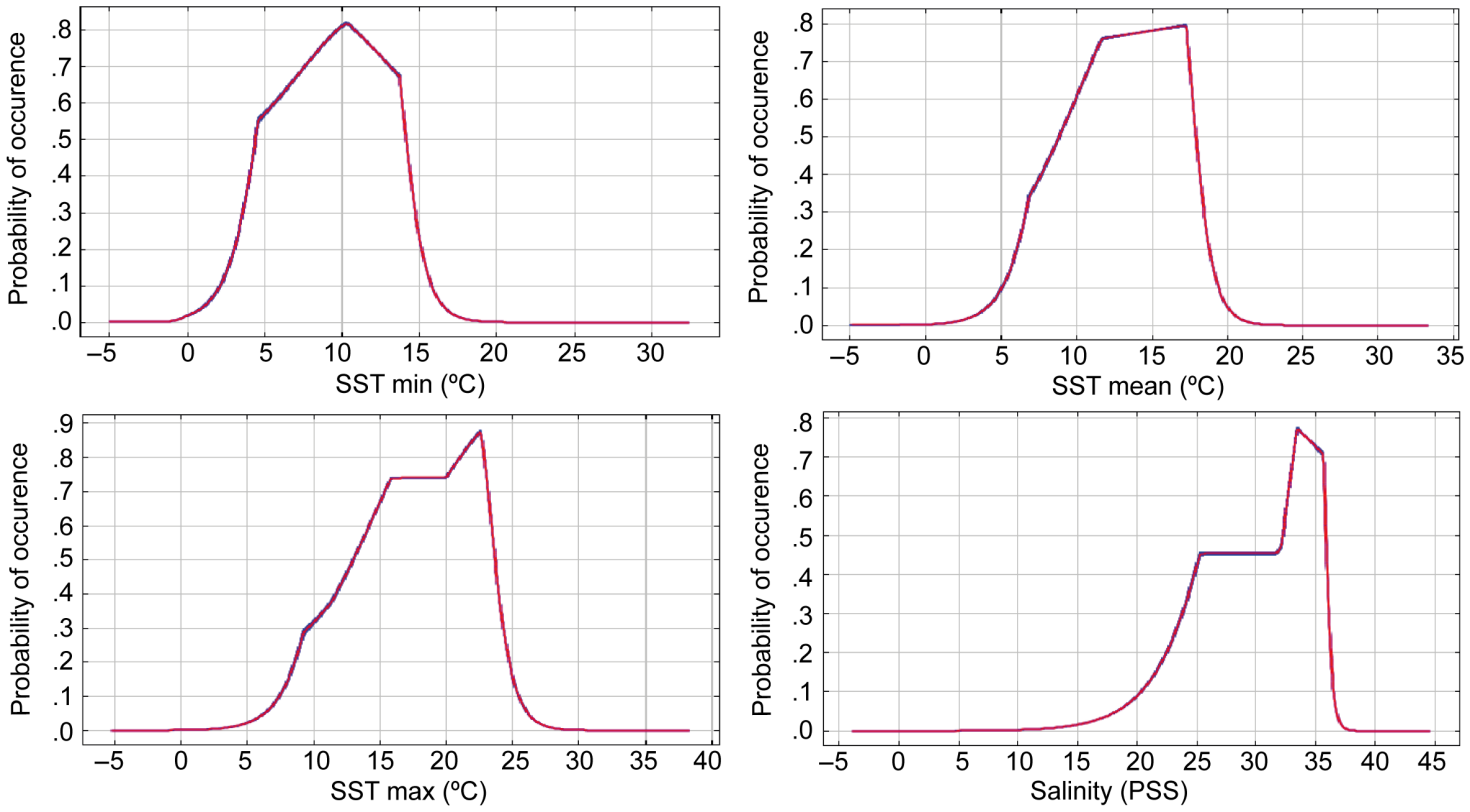
Predicted probability of occurrence of *Macrocystis pyrifera* of the variables of ${\mathrm{SST}}_{\min}$ (Sea Surface Temperature minimum), ${\mathrm{SST}}_{\text{mean}}$ (Sea Surface Temperature mean), ${\mathrm{SST}}_{\max}$ (Sea Surface Temperature maximum), and salinity for the present model (subset predictors). The mean of the 100 replicates is shown in red and the mean ± one standard deviation is shown in blue.

Focusing on future projection, the probability of global occurrence of *M*. *pyrifera* was analyzed using four variables. The independently predicted habitat suitability of each variable with the data extracted from the global occurrences is shown in Figure 2. For ${\mathrm{SST}}_{\min}$, the maximum probability of occurrence was observed around 10°C and a range of values between −1 and 18°C. For ${\mathrm{SST}}_{\text{mean}}$, the maximum values were around 12 and 18°C, and no probability of occurrence of *M*. *pyrifera* was obtained below 2°C and above 22°C. For ${\mathrm{SST}}_{\max}$, the curve reached the highest probability between 16 and 23°C. Temperature ranges oscillated between 15 and 20°C where at ${\mathrm{SST}}_{\min}$ the curve shifted toward temperatures and at ${\mathrm{SST}}_{\max}$ shifted to higher values. The maximum probability values for salinity ranged from 33 to 36 PSS.

**FIGURE 3:**
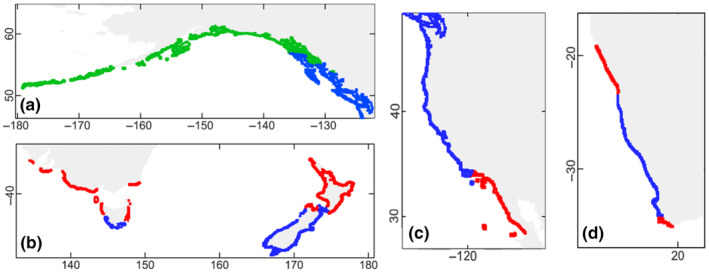
The suitable habitat modeled for *Macrocystis pyrifera* with the variables of ${\mathrm{SST}}_{\min}$, ${\mathrm{SST}}_{\text{mean}}$, ${\mathrm{SST}}_{\max}$, and salinity. Different parts of the world are represented: (a) North‐West Pacific (Alaska/Canada), (b) Southeast Indian and Southwest Pacific (Australia/New Zealand), (c) North‐West Pacific (EU/Mexico), and (d) Southeast Atlantic (South Africa). The figure compares the distributions obtained in this model (subset predictors) with the future scenario 8.5 of 2090–2100, where the conserved distribution of the suitable habitat is shown in blue, the lost in red, and the gained in green. The distribution of habitat suitability was enlarged in thickness for better visualization.

Distributional ranges were calculated for all areas where the habitat suitability of *M*. *pyrifera* was modeled (Table 2). A decrease in the range of suitable habitat was observed on coastlines worldwide (Southeast Atlantic, Southeast Indian, Northeast, Southeast, and Southwest Pacific) when we compared the ranges of the different models and projections. The greatest loss of habitat suitability was observed for the RCP8.5 scenario across a broad range of latitudes (Figure 3). Habitat losses were concentrated in the low‐latitude sectors, which corresponded to the equatorward limits of the distribution of *M*. *pyrifera* in the Northern and Southern Hemispheres. However, an increase in the range of suitable habitat was observed at higher latitudes in the Northeast Pacific Ocean (Alaska and Canada). A minor change was observed under scenarios RCP2.6, 4.5, and 6.0 (Figures S3–S5).

**TABLE 2:** Latitudes degrees of the maximum range in the model distribution of *Macrocystis pyrifera* for the present (all predictors), present (subset predictors), and the RCPs 2.6, 4.5, 6.0, and 8.5 scenarios for 2090–2100.

Coastal regions	Range limit	Present	Present projection	2.6	4.5	6.0	8.5
Northeast Pacific	Upper	58.25° N	56.83° N	60.13° N	60.50° N	61.16° N	61.33° N
Northeast Pacific	Lower	27.58° N	28.17° N	28.83° N	29.58° N	33.08° N	33.08° N
Southeast Pacific	Upper	−13.33° S	−10.75° S	−11.67° S	−24.75° S	−25.42° S	−27.83° S
Southeast Pacific	Lower	−56.08° S	−56.08° S	−56.08° S	−56.08° S	−56.08° S	−56.08° S
Southeast Atlantic	Upper	−17.33° S	−18.75° S	−19.58° S	−20.84° S	−22.17° S	−23.50° S
Southeast Atlantic	Lower	−34.83° S	−34.83° S	−34.75° S	−34.50° S	−34.25° S	−34.08° S
Southeast Indian	Upper	−36.83° S	−34.58° S	−35.92° S	−36.09° S	−36.83° S	−42.17° S
Southwest Pacific	Upper	−34.33° S	−34.33° S	−35.97° S	−38.75° S	−39.51° S	−40.58° S

The spatial change in habitat suitability was also observed when calculating the total suitable area in km^2^ (Table 3). The difference can be seen by comparing the present model with eight variables and the projection model with four variables. By reducing the number of environmental variables and focusing only on SST and salinity, an increase in the area of habitat suitability was observed in all geographical areas. Regarding the projections, an increase was observed in the Northeast Pacific and Arctic, doubling the area in the RCP8.5 scenario compared to today. The increase in area follows the predicted shift in habitat suitability to higher latitudes toward a region with an extensive coastline (Northwestern Canada and Alaska). A similar pattern was observed for the Southeast Pacific. Although *M*. *pyrifera* now reaches the largest possible continental range (Patagonia), we observed an increase in the habitat suitability area. The increase was not reflected in the area (km^2^)—which remained stable in the present and future projections—as the area gained at high latitudes was compensated by losses at low latitudes (Peru and Northern Chile). In Africa and Australia/New Zealand, the compression of the geographic range is reflected by the decrease in habitat suitability.

**TABLE 3:** Total suitable area (km^2^) in the model distribution of *Macrocystis pyrifera* for the present (all predictors), present (subset predictors), and the RCPs 2.6, 4.5, 6.0, and 8.5 scenarios for 2090–2100.

Coastal regions	Present (km^2^)	Present projection (km^2^)	2.6 (km^2^)	4.5 (km^2^)	6.0 (km^2^)	8.5 (km^2^)
Northeast Pacific	190,096	204,554	287,426	316,339	373,225	431,311
Southeast Pacific	154,635	240,267	240,095	230,627	232,950	252,141
Southeast Atlantic	28,912	28,999	26,848	23,836	20,824	17.554
Southeast Indian/Southwest Pacific	116,946	136,913	123,745	99.048	93,024	60,928
Total	490,589	610,733	678,114	669,850	720,023	761,934

The above results refer to areas where *M*. *pyrifera* is currently found. However, a new suitable habitat was found in our projection model for the Northeast Atlantic, a location currently not occupied by giant kelp. It is important to note that this habitat suitability was predicted in the projection model after a very large latitudinal expansion of suitable habitat, of approximately 20°, in the RCP8.5 scenario (Table S1; Figure S6). Regarding the suitable habitat area for *M*. *pyrifera*, in Europe, it was more than 10 times greater between the present projection and the RCP8.5 scenario.

**FIGURE 4:**
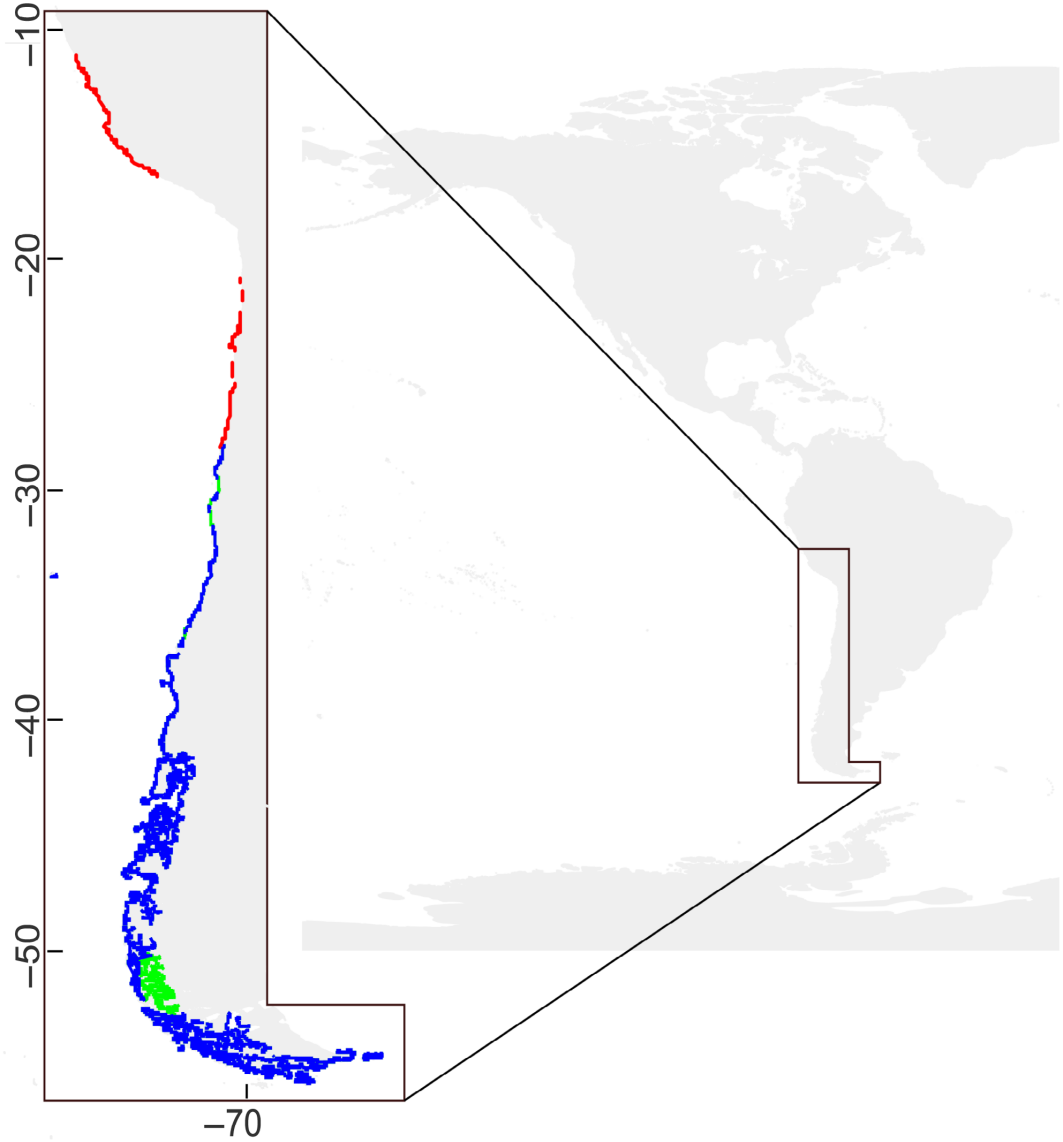
The suitable habitat modeled for *Macrocystis pyrifera* with the variables of ${\mathrm{SST}}_{\min}$, ${\mathrm{SST}}_{\text{mean}}$, ${\mathrm{SST}}_{\max}$ and salinity for the Southeast Pacific. The figure compares the distributions obtained in this model (subset predictors) with the future RCP8.5 scenario for 2090–2100, where the conserved distribution of the suitable habitat is shown in blue, the lost in red, and the gained in green. The habitat suitability distribution was enlarged in thickness for better visualization.

The results of the model for the Southern Pacific coast under the extreme RCP8.5 scenario showed a marked loss of habitat suitability along the coast (Figure 4). Suitable habitat was lost in its entirety along Peru and a large part of Northern Chile, resulting in a latitudinal range contraction of 17.08° S (Table 2). We observed the same pattern under RCP4.5 and RCP6.0, with suitable habitat conditions along the Peruvian coast predicted only under the RCP2.6 scenario (Figures S3–S5). It should be noted that the suitability of the modeled habitat was not continuous along the coastline. Our modeling indicates an area between Southern Peru (Arequipa, 16.4° S) and Northern Chile (Arica, 18° S) where no suitable habitat is observed (see Figure 4). Regarding the conserved distribution of habitat suitability, it remained stable in central and Southern Chile. Finally, an increase in habitat suitability was observed in Patagonia and two zones in central Chile. The expansions under the model offset the area lost in Peru and Northern Chile; therefore, it was not ultimately reflected in the total km^2^ of habitat suitability in the Southeast Pacific (Table 3). In fact, the suitable area increased slightly under the RCP8.5 scenario mainly due to the increase in the suitable area along the Fjordland of Chilean Patagonia where the coastal area is much larger than in the northern part.

## 4 DISCUSSION

### 4.1 Environmental predictors of the *Macrocystis pyrifera* SDM

Our global distribution model of *M*. *pyrifera* performed with good accuracy by fitting the model to the observed occurrences using a limited set of environmental variables, which were retained to project the model in the future. The model was also capable of accurately fitting the known distribution range of *M*. *pyrifera* based on the most thorough review of the group (Graham, Vasquez, & Buschmann, 2007). The ${\mathrm{SST}}_{\min}$ was the variable with the highest contribution to the global SDM of *M*. *pyrifera*, in agreement with the results of other models of distribution of giant kelp (Jayathilake & Costello, 2020; Martínez et al., 2018). The second variable was ${\mathrm{SST}}_{\text{mean}}$, confirming the importance of temperature for the global distribution of giant kelp, together with its role as a determining factor for local population declines (Butler et al., 2020; Wernberg et al., 2010). In terms of temperature, ${\mathrm{SST}}_{\max}$ was the third most important variable in terms of contribution to the model with a similar value to the ${\mathrm{SST}}_{\text{mean}}$ (20.4%). The importance of ${\mathrm{SST}}_{\max}$ is consistent with the sensitivity of *M*. *pyrifera* to the increase of temperature and extreme thermal events, such as heat waves, which have been shown to decimate local populations acutely exposed to them (Cavanaugh et al., 2019; Wernberg et al., 2010). Regarding the global distribution of *M*. *pyrifera*, low‐latitude limits were always associated with warmer waters, which are associated with decreased nutrient concentration, limited propagule survival, and competition with more tolerant species (Edwards & Hernandez‐Carmona, 2005; Hernandez‐Carmona et al., 2000; Ladah et al., 1999).

The projection model showed that the SST variables followed a Gaussian distribution when calculating how they impacted the global probability of occurrence of *M*. *pyrifera*. Our ${\mathrm{SST}}_{\text{mean}}$ curve coincides with the global distribution of the temperature range from 4 to 20°C (Schiel & Foster, 2015). Temperature is highly variable in different giant kelp populations worldwide, but there is agreement on a critical upper threshold—of 19–21°C—above which growth, gametogenesis, fertilization, and survival begin to be affected, for example, Southern California, Australia, and New Zealand (Butler et al., 2020; Cavanaugh et al., 2019; Deysher & Dean, 1986; Hay, 1990; North et al., 1986). Furthermore, rapid tissue degradation occurs when floating *Macrocystis* are exposed to temperatures above 20°C; hence, impacting dispersal by rafting (Rothäusler et al., 2009). The upper temperature threshold in the literature agrees well with the maximum ${\mathrm{SST}}_{\text{mean}}$ value above which our model did not allow a probability of occurrence of *M*. *pyrifera* (Figure 2). In sharp contrast, some populations at the equatorward limit of the distributional range, in populations such as San Diego and Baja California, have experienced SST of up to 24–26°C (North et al., 1986; Rosenthal et al., 1974). These populations at the edge of the range appear to be genetically distinct (Assis et al., 2023), and coincide with the observed threshold of ${\mathrm{SST}}_{\max}$ in our study. On the other hand, for populations located at higher latitudes, such as in Southern Chile, SST higher than 15–17°C explain the high mortality of adults observed during summer (Buschmann et al., 2004, 2014). The upper bound of the thermal limit for these populations in Southern Chile coincides with the maximum ${\mathrm{SST}}_{\min}$ where the probability of suitable habitat for *M*. *pyrifera* is still predicted. Therefore, the global temperature distribution range coincides with the ${\mathrm{SST}}_{\text{mean}}$, the thresholds in populations at low latitudes that are adapted to higher temperatures coincide with the ${\mathrm{SST}}_{\max}$ limit. Similarly, populations at higher latitudes that inhabit waters with lower temperatures coincide with our ${\mathrm{SST}}_{\min}$. our study encompasses all these local adaptations and the thresholds of the different populations when considering occurrences at the global level. In addition, according to the probability of occurrence estimated in our model, the thermal tolerance of *M*. *pyrifera* are in excellent agreement with a similar SDM recently published by (Assis et al., 2023).

${\mathrm{SST}}_{\min}$ and ${\mathrm{SST}}_{\text{mean}}$ are highly correlated with areas of intense coastal upwelling, which are associated with extensive and persistent *M*. *pyrifera* stands (Broitman & Kinlan, 2006; Graham, Vasquez, & Buschmann, 2007; Narayan et al., 2010). Around upwelling centers, a strong negative correlation is observed between SST and nutrients concentration, especially nitrate, two variables that can strongly affect the populations of *Macrocystis* (Hernandez‐Carmona et al., 2001; Nielsen & Navarrete, 2004; Zimmerman & Kremer, 1984). The responses of giant kelp to the variation of temperature and nitrate are complex to elucidate following their inverse correlation (North et al., 1986; Schiel & Foster, 2015). In our model, no correlation was found between SST and nitrate. In this regard, it should be noted that we considered the entire global coastline and therefore areas that include upwelling and non‐upwelling zones and where other relations between temperature and nitrate may prevail. Of the two nutrients considered in the study, phosphate stands out for its high contribution to the model compared to the low value of nitrate. Phosphate is essential for macroalgae development as it is a structural component of key macromolecules such as nucleic acids, phospholipids, ATP/ADP, and could be a limiting factor on the growth of adult *M*. *pyrifera* (Manley & North, 1984; Mizuta et al., 2003). In our study, the importance of phosphate was reflected in an 18.8% contribution to the global habitat suitability for giant kelp. On the other hand, nitrate is the nutrient that has received the greatest attention to understand *M*. *pyrifera*—particularly growth—yet giant kelp forests use less than 5% of the nitrate that reaches them, extracting much of it from other sources, such as ammonium from epibionts (Fram et al., 2008; Reed & Brzezinski, 2009; Rodriguez et al., 2016; Zimmerman & Kremer, 1986). In our model, the concentration of nitrate contributed with a very low percentage (2.1%) to explain the habitat suitability of *M*. *pyrifera* indicating that, on a global scale, this nutrient does not appear to be a determining factor for its distribution. The weak contribution of nitrate to the model may result from the scale of the study, as we used a global environmental dataset with limited spatial resolution (Assis, Tyberghein, et al., 2018). Hence, it is not possible to discern the locations or times of the year where nitrate may be a limiting factor for the growth of *M*. *pyrifera*. Other important predictors recognized in the study of Jayathilake and Costello (2020) for *M*. *pyrifera* were distance to land and wave height. We incorporated the former when creating the coastal layer mask in our study, while the latter was not incorporated although it is known to be important for giant kelp settlement and interannual variation in the cover of the kelp beds (Dayton & Tegner, 1984; Reed et al., 2011). It should be noted that, to date, future projections of significant wave height are still of a very large spatial scale, precluding their use in our model (Badriana & Lee, 2021).

### 4.2 Global distribution of habitat suitability

In terms of the spatial distribution of habitat suitability, we observed range contractions, especially under the extreme 2090–2100 RCP8.5 scenario, at all equatorial range edges (Mexico, United States, Peru, Northern Chile, South Africa, Australia, and New Zealand), a range limit associated with warm oligotrophic waters (Graham, Vasquez, & Buschmann, 2007). Range contractions could also be related to the thermal physiological limit of *M*. *pyrifera* mentioned above, which can lead to severe reductions in canopy biomass and a decrease in blade elongation rates (Rodriguez et al., 2016). In Australia, the disappearance of giant kelp forests has already been predicted if SST continues to increase (Martínez et al., 2018; Wernberg et al., 2011). In general, the retreat or disappearance of kelp populations usually occurs within the limits of the distribution range where tolerance to multiple abiotic factors is exceeded (Wernberg et al., 2010). However, an increase in the area of suitable habitat for giant kelp was observed in the model at high latitudes, for example, Alaska/Canada and Patagonia. This increase is bordered by the Arctic environment and coincides with future loss of habitat suitability of cryotolerant macroalgae belonging to that region (Bringloe et al., 2022). This extension of the range and area of habitat suitability can lead to an extension of these highly productive macroalgal forests and the associated faunal biodiversity and ecosystem services that would result from these ecosystems (Bayley et al., 2021; Cuba et al., 2022). However, we consider treating these results with caution since the projection was made only using temperature and salinity. The limiting variables described for the giant kelp distribution at high latitudes (e.g., Northern California, Southern Chile) are low solar isolation and wave action, which were not considered in the model (Buschmann, 1992; Buschmann et al., 2014; Foster & Schiel, 1985; Graham et al., 1997; Huovinen et al., 2020; Palacios et al., 2021). Despite that, the increase in total area observed in our study coincides with the expansion of algal forests into polar and subpolar areas and this expansion was reflected in the total area of suitable global habitat (Duarte et al., 2022). Our results are in line with evidence that the suitable habitat for giant kelp during the Last Glacial Maximum (LGM) period was smaller toward higher latitudes in the Northern Hemisphere. On the contrary, habitat suitability in lower latitudes (Mexico) was the only region that saw an increase between the LGM and the present (Assis et al., 2023). Together with insights from the study of Assis et al. (2023), our results suggest a poleward expansion of *M*. *pyrifera* under multiple scenarios of increasing greenhouse concentrations in the atmosphere.

Finally, the suitability of the modeled habitat in areas far removed where giant kelp currently inhabits, as is the case in Europe, highlight the caveats necessary to interpret the SDM results. Habitat suitability around Europe was not modeled when we considered all predictors, but it appeared when considering our restricted set of predictors: only SST and salinity (Table S1; Figure S6). Therefore, the concentration of key nutrients, which as discussed above, are highly correlated with SST in upwelling regions, may play a limiting role that is captured in the full model. It is interesting to note that between the 1950s and 1970s, the introduction of *Macrocystis* was considered for European aquaculture and finally was not carried out following social pressure; the attempt provided evidence that individuals could survive on British coasts (Boalch, 1980). Moreover, at the time it was considered possible that this species could colonize the European Atlantic coast, from Spain to Norway, with unpredictable consequences (Boalch, 1980), which is in good agreement with the results of our model.

When considering the area of habitat suitability by our modeling study, we highlight that it is an approximation of the actual area that *M*. *pyrifera* could inhabit due to the coarse spatial resolution of the variables used (9.2 km), together with other abiotic factors not taken into account, for example, waves, ice, or rocky substrate. For example, the expansion of giant kelp to higher latitudes is expected to follow the increase in the number of days of open water (free from ice), thus a broader bathymetric range (Castro de la Guardia et al., 2023). *M*. *pyrifera* has limited dispersal through spores (Reed et al., 2004); yet, it can raft and maintain population connectivity over extremely long distances, at least in the Southern Hemisphere (Batista et al., 2018). As in the projections, other factors could influence the possible future distribution of giant kelp, such as an intensification of coastal upwelling, which will mitigate the increase of SST, thus dampening the effects of global change on kelp populations (Bakun, 1990; Narayan et al., 2010; Varela et al., 2018). Populations adapted to the low pH levels experienced under strong upwelling conditions have been observed to produce more eggs through increased fertilization success (Hollarsmith et al., 2020). In addition, ecological processes such as competition or herbivory were not taken into account. For example, in Patagonia, Argentina, the invasion of a giant kelp forest by a non‐native kelp *Undaria pinnatifida* reduced the richness, abundance, and diversity of the accompanying fauna (Raffo et al., 2009). Also, a warming ocean is driving the poleward expansion of tropical herbivores that can overgraze a temperate kelp forest, reorganize benthic communities, and haste equatorial range–edge contractions (Vergés et al., 2014). The opposite situation can take place at higher latitudes. For example, prolonged warming has led to almost complete displacement of the kelp *Nereocystis luetkeana* by *M*. *pyrifera*, better adapted to the higher temperatures now prevalent in a location in central California (Schiel et al., 2004; Schiel & Foster, 2015). In addition to ecological effects, patterns of local adaptation among of different populations of giant kelp will also play an important role (Fernández et al., 2021). Future modeling could take into account the different morphotypes, *M*. *pyrifera* and *M*. *integrifolia*, as well as the potentially adaptive differences between populations of different hemispheres as suggested by recent whole‐genome studies along the eastern Pacific (Gonzalez et al., 2023). Despite the myriad of physical and biological processes that take place on local scales, temperature is consistently one of the most relevant variables to explain the global distribution of *M*. *pyrifera*. Hence, our parsimonious model provides a robust approximation to forecast changes in habitat suitability for this key habitat‐forming kelp species under IPCC climate projections.

### 4.3 Habit suitability along the Southeast Pacific

By focusing on the habitat suitability distribution along the Southeast Pacific, we highlight the complete loss of suitability in Peru and Northern Chile under the RCPs 4.5, 6.0 and 8.5 scenarios (Table 2, Figure 4). Currently, the species shows a fragmented distribution throughout the region, which has been proposed to be a long‐lasting effect of the unprecedented 1982–83 El Niño–Southern Oscillation (ENSO) event, which decimated local populations and had similar effects in Southern California (Arntz & Tarazona, 1990; Dayton & Tegner, 1984; Glynn, 1988). Extreme events are of special concern, as they can drive range contractions over short temporal scales; future projections predict an increase in ENSO magnitude under greenhouse warming (Cai et al., 2021). The observed discontinuity in *M*. *pyrifera* distribution between Peru and Northern Chile could be due to the extensive sandy beaches in that area or weak seasonal upwelling along the region (Assis et al., 2023; Blanco et al., 2001). The lack of availability of rocky habitat could be compounded by future climate scenarios for the region showing a decrease of upwelling‐favorable winds in summer, which could lead to lower nutrient concentrations and more frequent coastal warming conditions (Chamorro et al., 2021; Rykaczewski et al., 2015), both critical for giant kelp.

Along the Southeast Pacific, two different morphotypes of *Macrocystis* coexist, for example, *pyrifera* and *integrifolia*. Maximal growth rates for the latter were observed at low temperatures (8°C) (Buschmann et al., 2004; Macaya & Zuccarello, 2010). The long latitudinal extent of the coast of Chile harbors local adaptations to thermal conditions, with larger tolerance ranges in low‐latitude populations when compared to higher latitude ones (Buschmann et al., 2004). Specifically, in populations in Southern Chile, high mortality was associated with temperature higher than 15–17°C, in agreement with the maximum ${\mathrm{SST}}_{\min}$ value temperature for probability of occurrence of *M*. *pyrifera* in our study (Buschmann et al., 2014). Despite the risk of differential sensibility of the morphotypes to temperature extremes—that is, ENSO events—the loss of habitat suitability may be buffered in some areas through topographic intensification of coastal upwelling (Aravena et al., 2014; Broitman & Kinlan, 2006). Such local‐scale processes could create relic populations of crucial conservation and management targets (Assis et al., 2023; Lourenço et al., 2016).

Wild populations of *M*. *pyrifera* along the predicted extirpation area in Peru and Chile are currently under intense exploitation, which has followed an upward trend in recent years (SERNAPESCA, 2021). The harvested biomass is dried, ground, and exported for alginate extraction; thousands of people depend directly or indirectly on the activity (Avila‐Peltroche & Padilla‐Vallejos, 2020; Vásquez et al., 2014). In Northern Chile, an estimate of the value of kelp forests is 541 million USD$ based on direct harvesting, associated fisheries, their value in education, ecotourism, as a buffer for climate, and as a target of scientific studies (Vásquez et al., 2014). Our study predicts a loss of habitat suitability from its current equatorward range edge at ca. 12° S in central Peru to 27.83° S in Northern Chile. A total of 11,180 tons of *M*. *pyrifera* are currently harvested in the area showing a loss of habitat suitability in our model for Chile (annual average between 2012 and 2021, Figure S7), which is equivalent to one third of the value for the entire country (31,860.3 tons)(SERNAPESCA, 2021). The economic losses due to the loss of suitable habitat for giant kelp are worrying. However, such direct cost will further increase when other ecosystem services are considered; for example, 210 species are associated with kelp forests (in Chile), some of which are commercial fisheries, or may act as blue carbon (Cuba et al., 2022; Vásquez et al., 2014). In addition, the loss of habitat suitability observed in our study can spill beyond local fishers incomes into an emerging aquaculture industry of *M*. *pyrifera* in Peru and Chile (Boada Medina, 2021; Buschmann et al., 2014; Camus et al., 2019).

## 5 CONCLUSION

Ongoing climate change is changing the distribution of entire assemblages and is of particular concern when habitat‐forming species such as kelp are impacted. *M*. *pyrifera* is the most widely distributed kelp species, and temperature is the most important factor influencing its global distribution (Graham, Vasquez, & Buschmann, 2007). The results of our species modeling show large range shifts in the global distribution of *M*. *pyrifera* under future climate change scenarios, with ${\mathrm{SST}}_{\min}$ showing the largest contribution to the model predictions. Due to the importance of temperature and predicted global warming, it was expected to observe a loss of habitat suitability in the low‐latitude sectors of the geographic range. Future studies should consider adding other locally relevant variables to the model, such as wave climate or substrate. Incorporating such variables will be possible only when forecasts become available at relevant spatial resolutions; hence, allowing improvements to the modeled habitat suitability, and therefore potential distribution maps closer to the observed distribution. The implementation of management measures will be critical to the conservation of *M*. *pyrifera* populations and the future sustainability of these kelp forests. These measures are particularly relevant in areas where economic and social importance will be strongly influenced by the loss of *M*. *pyrifera* populations, as would be the case of Peru and Chile.

## AUTHOR CONTRIBUTIONS

**Daniel Gonzalez‐Aragon:** Conceptualization (lead); formal analysis (lead); investigation (lead); methodology (lead); project administration (lead); writing – original draft (lead); writing – review and editing (lead). **Marcelo M. Rivadeneira:** Conceptualization (equal); investigation (equal); methodology (equal); validation (equal); writing – review and editing (equal). **Carlos Lara:** Conceptualization (equal); funding acquisition (equal); investigation (equal); supervision (equal); visualization (equal); writing – original draft (equal); writing – review and editing (equal). **Felipe I. Torres:** Conceptualization (equal); methodology (equal); visualization (equal); writing – original draft (equal); writing – review and editing (equal). **Julio A. Vasquez:** Data curation (supporting); writing – review and editing (equal). **Bernardo R. Broitman:** Conceptualization (equal); funding acquisition (equal); supervision (equal); visualization (equal); writing – original draft (equal); writing – review and editing (equal).

## CONFLICT OF INTEREST STATEMENT

The authors declare that they have no conflict of interest.

### OPEN RESEARCH BADGES

This article has earned an Open Materials badge for making publicly available the components of the research methodology needed to reproduce the reported procedure and analysis. All materials are available at the occurrences of *Macrocystis pyrifera* used in the study, the coastal mask to cut the environmental predictors, and the environmental predictors used are available at: https://datadryad.org/stash/share/gAAAAPdP30UlRazQYRqsiMIH_dujGthRonrsY_e422U.

## Supporting information


Appendix S1.

## Data Availability

The occurrences of *Macrocystis pyrifera* used in this study, the coastal mask to cut the environmental predictors, and the environmental predictors used are available at https://datadryad.org/stash/share/gAAAAPdP30UlRazQYRqsiMIH_dujGthRonrsY_e422U.
