# Supplementary material for: A species distribution model of the giant kelp Macrocystis pyrifera: Worldwide changes and a focus on the Southeast Pacific
Source: Ecol Evol. 2024 Mar 1;14(3):e10901. doi: 10.1002/ece3.10901 (PMC10905252; doi:10.1002/ece3.10901)
Supplement: Supplementary file 1 — Appendix S1. [file ECE3-14-e10901-s001.pdf]

TABLE S1 Latitudes degrees of the maximum upper range and total suitable area (km^2) for the Northeast Atlantic in the model distribution of *M. pyrifera* for the present (all predictors), present (subset predictors) and the RCPs 2.6, 4.5, 6.0, and 8.5 scenarios for 2090-2100.

Coastal regions	Present	Present projection	2.6	4.5	6.0	8.5
Northeast Atlantic	-	52.24° N	53.25° N	54.17° N	54.92° N	71.17° N
Northeast Atlantic	-	28,397	63,420	87,174	100,513	288,028

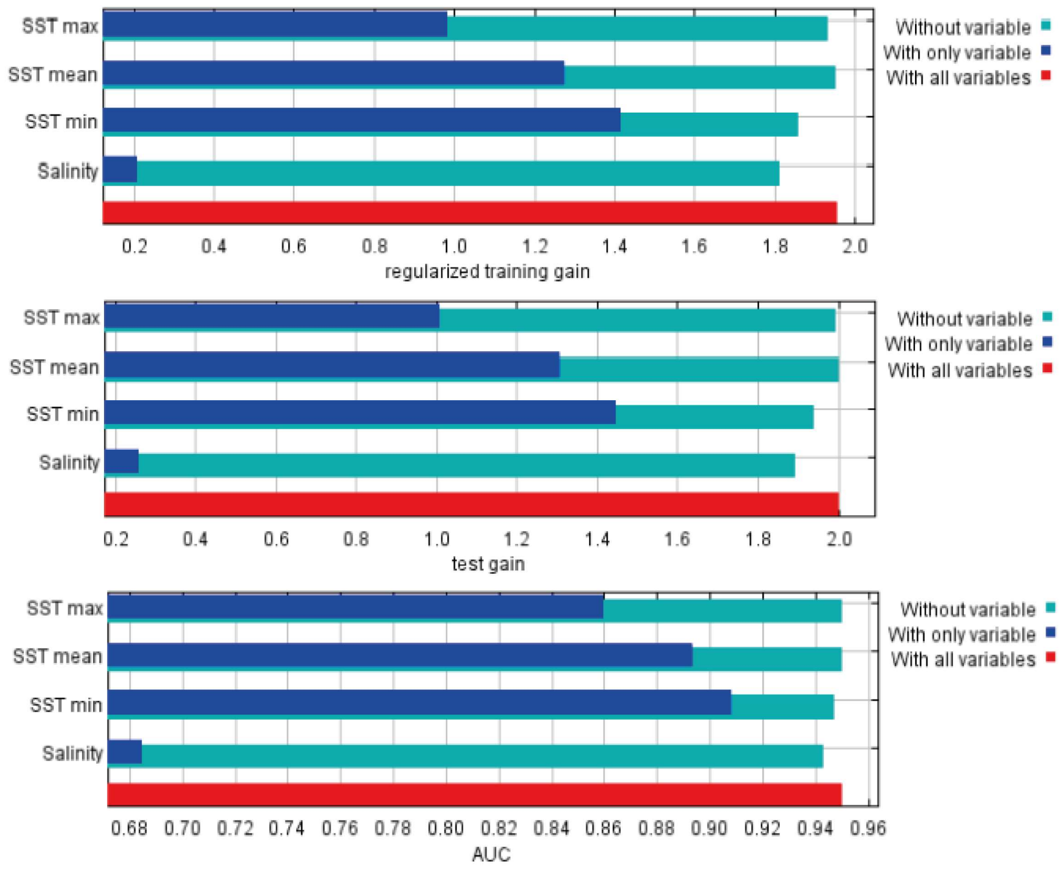

FIGURE S1 Jack-knife result of predictors variables for training gain (upper), test gain (middle), and AUC (down). The test with only the predictor is represented in blue, without the predictor in green, and with all variables in red.

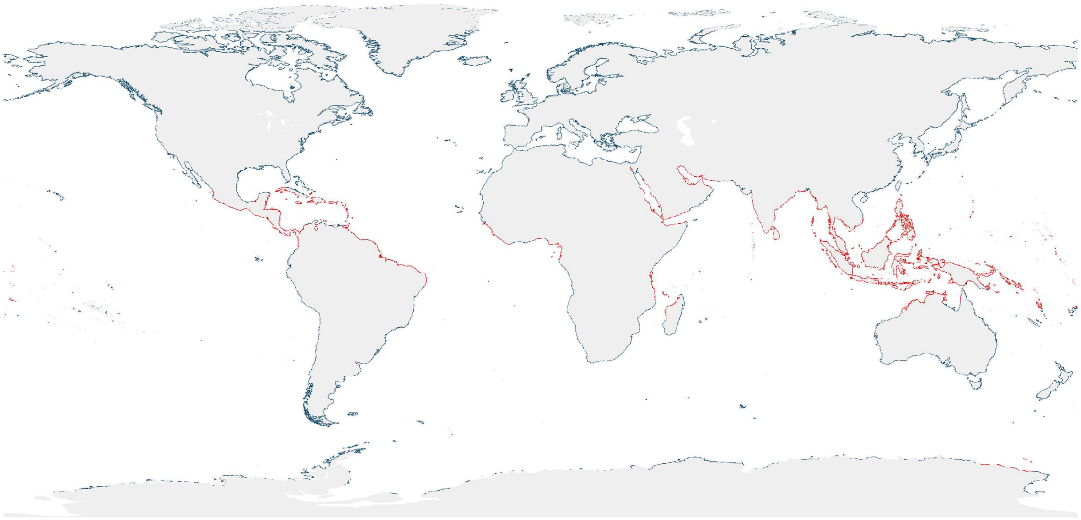

FIGURE S2 Global Multivariate Environmental Similarity Surface (MESS) analysis from Maxent comparing the present model (subset predictors) against RCP8.5 scenario for 2090-2100. Positive values are representing in blue and negative values in red.

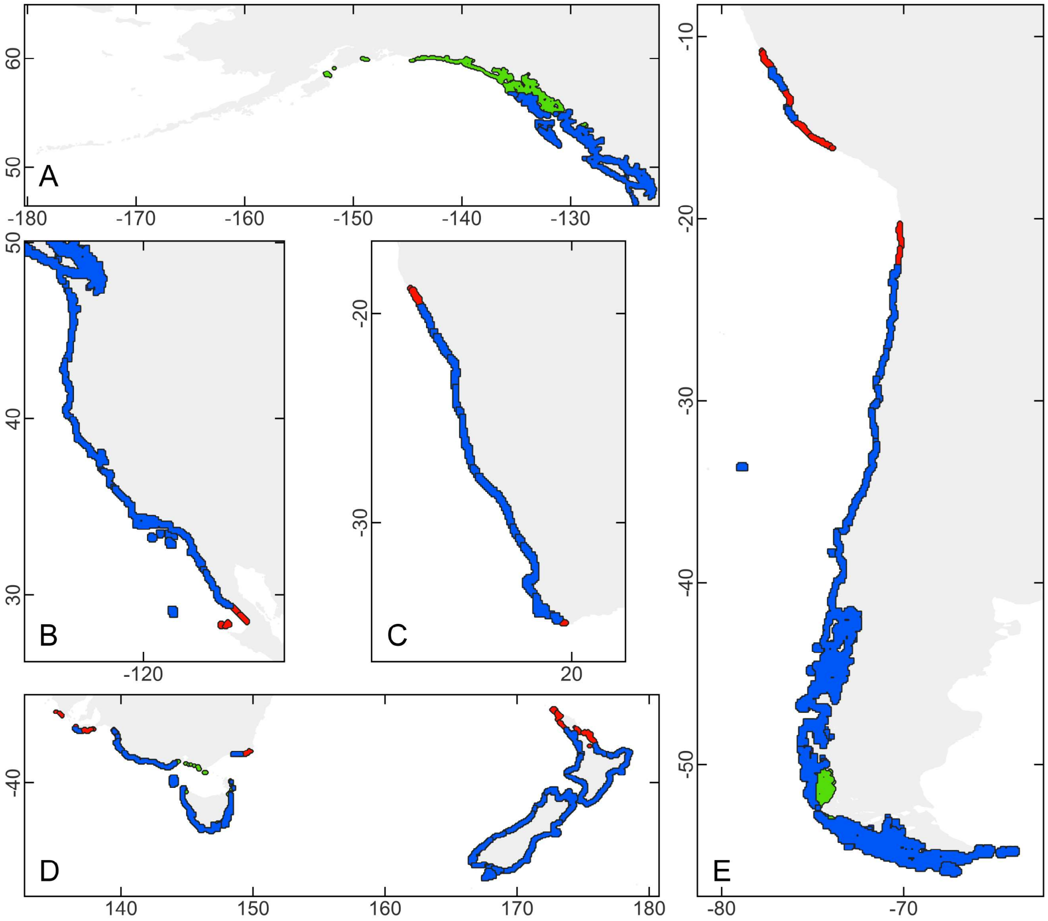

FIGURE S3 The suitable habitat modeled for *M. pyrifera* with the variables of SST_{min}, SST_{mean}, SST_{max}, and salinity. Different parts of the world are represented: (A) North-West Pacific (Alaska/Canada), (B) North-West Pacific (EU/Mexico), (C) Southeast Atlantic (South Africa), (D) Southeast Indian and Southwest Pacific (Australia/New Zealand), and (E) the Southeast Pacific (Peru/Chile). The figure compares the distributions obtained in the present model (subset predictors) with the future scenario 2.6 of 2090-2100, where the conserved distribution of the suitable habitat is shown in blue, the lost in red, and the gained in green. The distribution of habitat suitability was enlarged in thickness for better visualization.

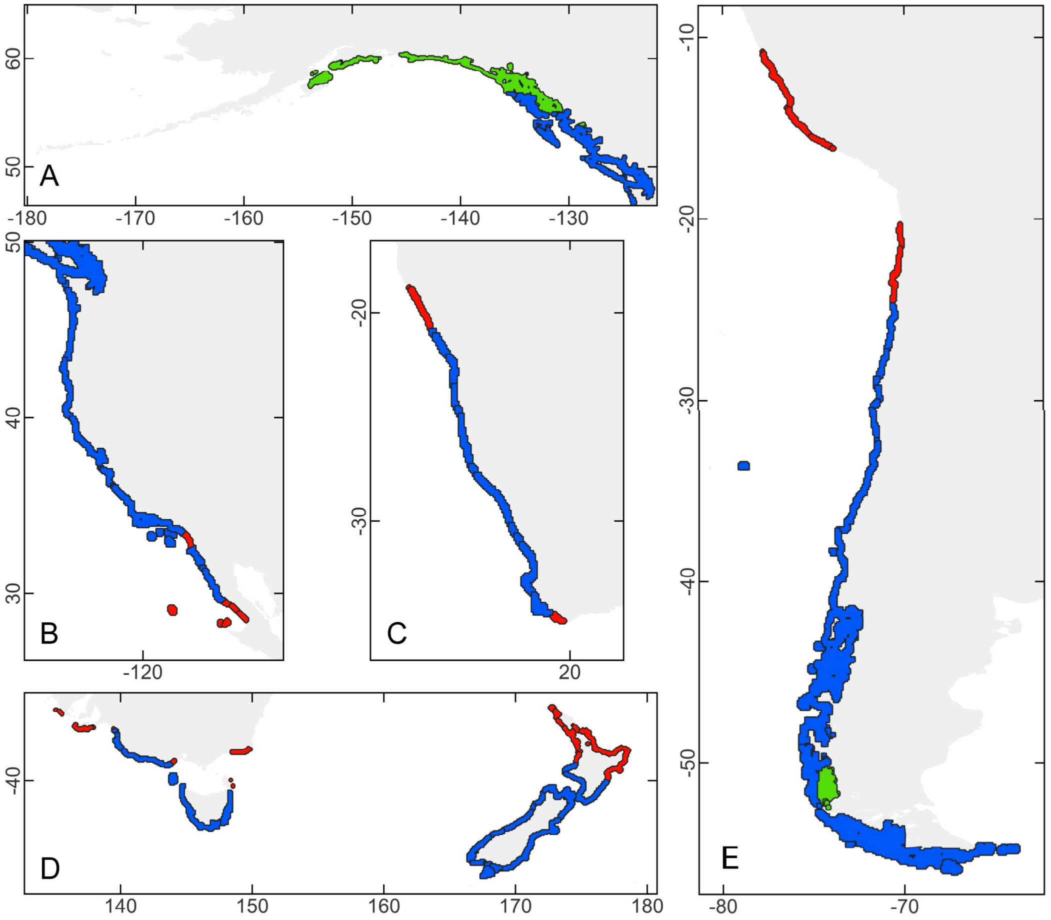

FIGURE S4 The suitable habitat modeled for *M. pyrifera* with the variables of SST_{min}, SST_{mean}, SST_{max}, and salinity. Different parts of the world are represented: (A) North-West Pacific (Alaska/Canada), (B) North-West Pacific (EU/Mexico), (C) Southeast Atlantic (South Africa), (D) Southeast Indian and Southwest Pacific (Australia/New Zealand), and (E) the Southeast Pacific (Peru/Chile). The figure compares the distributions obtained in the present model (subset predictors) with the future scenario 4.5 of 2090-2100, where the conserved distribution of the suitable habitat is shown in blue, the lost in red, and the gained in green. The distribution of habitat suitability was enlarged in thickness for better visualization.

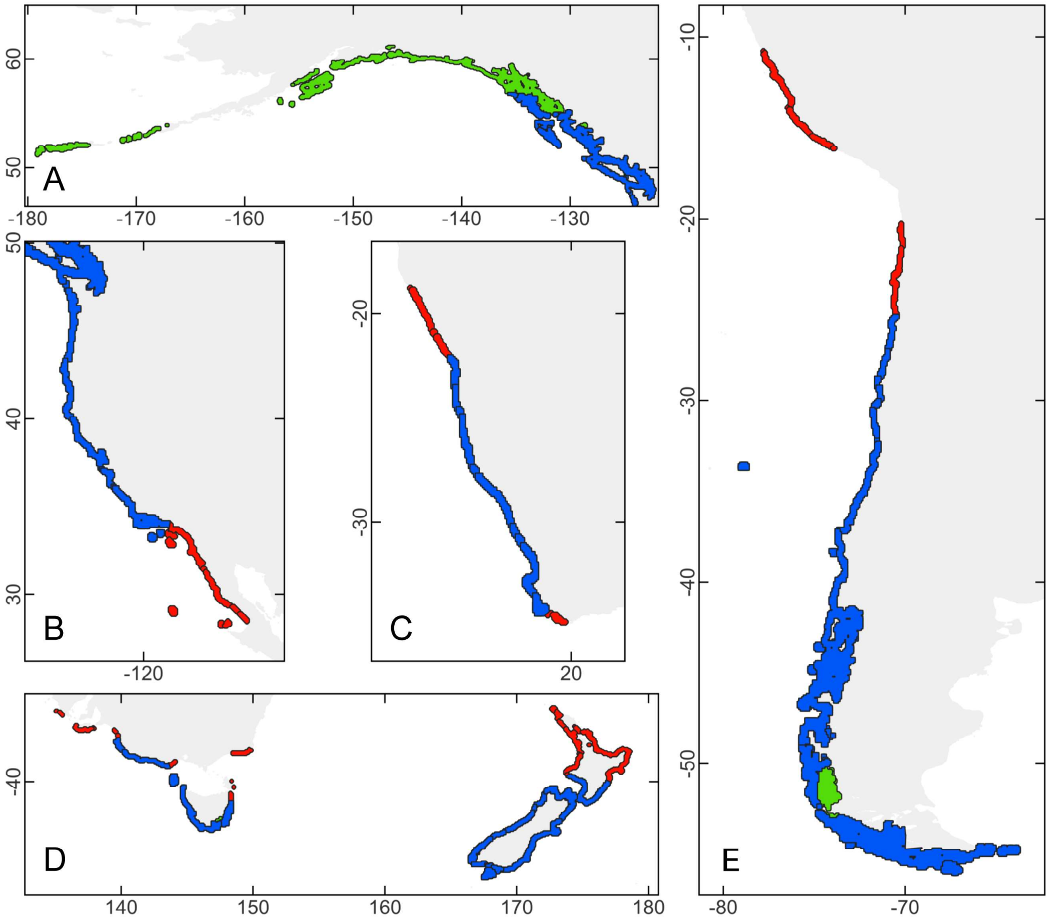

FIGURE S5 The suitable habitat modeled for *M. pyrifera* with the variables of SST_{min}, SST_{mean}, SST_{max}, and salinity. Different parts of the world are represented: (A) North-West Pacific (Alaska/Canada), (B) North-West Pacific (EU/Mexico), (C) Southeast Atlantic (South Africa), (D) Southeast Indian and Southwest Pacific (Australia/New Zealand), and (E) the Southeast Pacific (Peru/Chile). The figure compares the distributions obtained in the present model (subset predictors) with the future scenario 6.0 of 2090-2100, where the conserved distribution of the suitable habitat is shown in blue, the lost in red, and the gained in green. The distribution of habitat suitability was enlarged in thickness for better visualization.

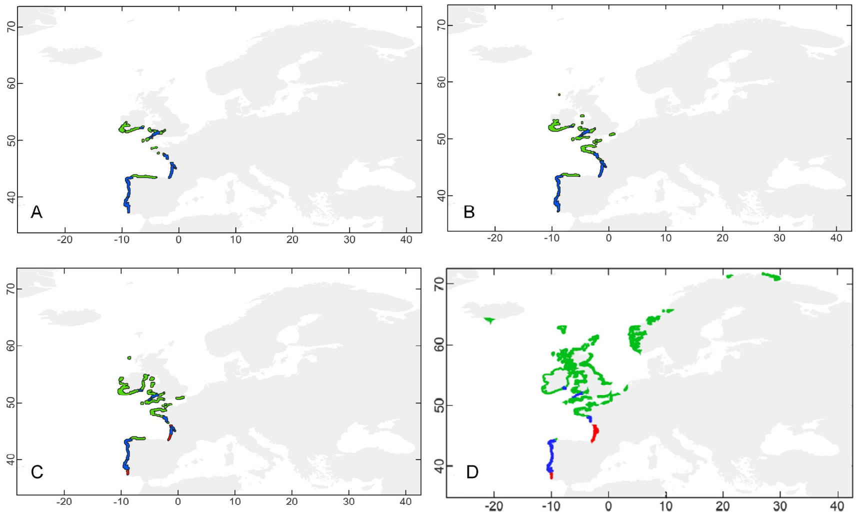

FIGURE S6 The suitable habitat modeled for *M. pyrifera* with the variables of SST_{min}, SST_{mean}, SST_{max}, and salinity for the North-East Atlantic (Europe).The figure compares the distributions obtained in the present model (subset predictors) with the future RCP2.6 (A), RCP4.5 (B), RCP6.0 (C), and RCP8.5 (D) scenarios for 2090-2100, where the conserved distribution of the suitable habitat is shown in blue, the lost in red and the gained in green. The habitat suitability distribution was enlarged in thickness for better visualisation.

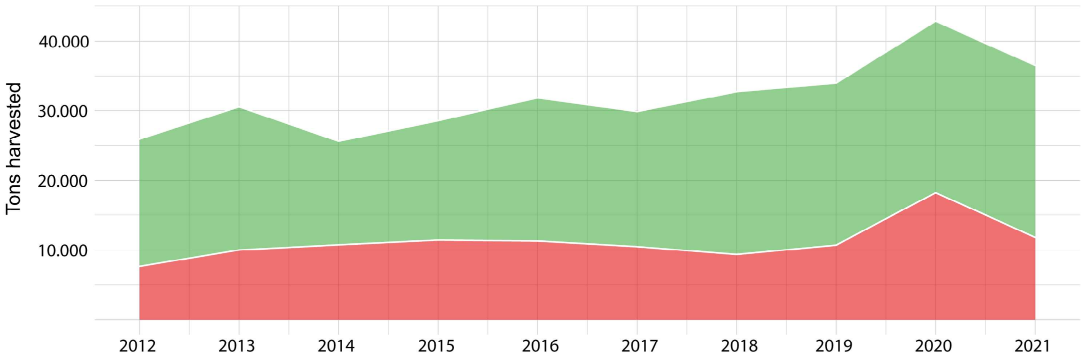

FIGURE S7 Total tons of *M. pyrifera* harvested in Chile between 2012 to 2021. The part in red represent the tons harvested in the coves where our model indicates that habitat suitability will be lost and in green are the tons harvested where habitat suitability will be conserved.
